# N-Terminal Processing and Modification of Ciliary Dyneins

**DOI:** 10.3390/cells12202492

**Published:** 2023-10-20

**Authors:** Miho Sakato-Antoku, Jeremy L. Balsbaugh, Stephen M. King

**Affiliations:** 1 Department of Molecular Biology and Biophysics, University of Connecticut Health Center, 263 Farmington Avenue, Farmington, CT 06030-3305, USA; mantoku@uchc.edu; 2 Proteomics and Metabolomics Facility, University of Connecticut, 75 North Eagleville Road, Storrs, CT 06269, USA; jeremy.balsbaugh@uconn.edu

**Keywords:** acetylation, axoneme, *Chlamydomonas*, cilia, degron, dynein, microtubule, N-end rule

## Abstract

Axonemal dyneins are highly complex microtubule motors that power ciliary motility. These multi-subunit enzymes are assembled at dedicated sites within the cytoplasm. At least nineteen cytosolic factors are specifically needed to generate dynein holoenzymes and/or for their trafficking to the growing cilium. Many proteins are subject to N-terminal processing and acetylation, which can generate degrons subject to the ^Ac^N-end rule, alter N-terminal electrostatics, generate new binding interfaces, and affect subunit stoichiometry through targeted degradation. Here, we have used mass spectrometry of cilia samples and electrophoretically purified dynein heavy chains from *Chlamydomonas* to define their N-terminal processing; we also detail the N-terminal acetylase complexes present in this organism. We identify four classes of dynein heavy chain based on their processing pathways by two distinct acetylases, one of which is dependent on methionine aminopeptidase activity. In addition, we find that one component of both the outer dynein arm intermediate/light chain subcomplex and the docking complex is processed to yield an unmodified Pro residue, which may provide a setpoint to direct the cytosolic stoichiometry of other dynein complex subunits that contain N-terminal degrons. Thus, we identify and describe an additional level of processing and complexity in the pathways leading to axonemal dynein formation in cytoplasm.

## 1. Introduction

Cilia are motile, sensory, and secretory organelles that protrude into the extracellular space [1]. Motile cilia are usually constructed around nine outer doublet microtubules with a central pair of singlet microtubules that together form the axoneme. Signals from the central pair propagate through radial spokes and alter the activity of dynein motors that are arranged as inner and outer rows of axonemal dynein arms. These motors generate inter-doublet sliding [2]. In order to obtain a ciliary beat, regions of active sliding propagate along the ciliary length, and doublets that are actively sliding must switch to generate principal and reverse bends. This switching needs to operate at a rate commensurate with the beat frequency that, in some systems, can exceed 100 Hz. Ciliary motility can power the movement of individual cells and also move fluids across surfaces. Dynein-based ciliary motility dates to before the last eukaryotic common ancestor and is found throughout the eukaryotes except for a few select phylogenetic groups such as red algae and angiosperms [3]. Furthermore, an additional dynein motor, more closely related to canonical cytoplasmic dynein, is required for retrograde intraflagellar transport (IFT), which is necessary for building cilia and maintaining their integrity. In mammals, defects in ciliary dynein motors lead to developmental defects, infertility, situs inversus, and severe bronchial problems [4,5]; more broadly, defective ciliary motility has been linked to congenital heart disease [6] and some forms of epilepsy [7,8,9].

Dyneins are built around the large, heavy chain (HC) motor units that consist of ~4500 residues and are members of the AAA+ family of ATPases [10]. These motors have a large N-terminal region involved in the assembly of the holoenzyme, followed by a linker domain that traverses across a ring of six AAA+ domains (AAA1–AAA6). The microtubule-binding site is located at the tip of an extended coiled coil that emanates from AAA4 [11,12]. Changes in linker domain orientation and microtubule affinity are driven by ATP binding and hydrolysis at AAA1 [13,14] and modified by nucleotide binding to AAA2, AAA3, and/or AAA4 [15]. The outer dynein arm and inner-arm I1/f contain two or (in some cases) three different HCs that are associated with a series of intermediate and light chains (ICs and LCs) to form a stable complex. In addition, the inner-arm system includes a series of monomeric HC motors, each associated with an actin monomer and other accessory components. These multi-protein assemblages interact with docking structures to bind precise locations on the doublet microtubules and are also involved in motility regulation in response to changes in various parameters such as Ca^2+^ concentration, phosphorylation status, and redox poise [16]. Synthesis of axonemal dyneins occurs in the cytoplasm and is highly complex; to date, nineteen specific cytoplasmic factors have been identified as involved in this process [17,18,19]. 

Many proteins are subject to post-translational alterations through multiple enzymatic routes. These usually reversible reactions control a vast array of biological activities and processes. Dedicated enzymes are used to both add and remove the alterations. In some cases, the modifications act directly as a switch to change some feature or activity of the target protein, whereas in others, additional proteins read the modified output and transmit that information, thereby altering downstream biological responses. Post-translational modifications of some dynein proteins have been identified previously. Several axonemal dyneins are controlled by phosphorylation, although the modified regulatory sites identified to date are located within ICs and/or LCs rather than the motor units themselves [20,21,22]. In *Chlamydomonas*, phosphorylation of the outer-arm dynein α HC and several monomeric inner-arm HCs has been demonstrated [23,24,25]; one mass spectrometry study also found a single phosphorylation site within the outer-arm γ HC [26]. However, the functional consequence(s) of these HC modifications are unknown. To our knowledge, there have been no reports of dynein proteins being subject to other types of post-translational modification.

Protein N-terminal acetylation occurs throughout the eukaryotes [27] and is involved in the control of protein stability and folding, the formation of new binding interfaces, subcellular localization, and potentially other processes such as directing available subunit stoichiometry [27,28,29]. For example, the regulation of tropomyosin–actin interactions in *Saccharomyces cerevisiae* is controlled by the N-terminal acetylation of Tpm1 [30]. This very common modification alters electrostatics by removing the +1 charge at the protein N-terminus. In eukaryotes, there are multiple N-terminal acetyltransferase complexes (NatA-NatH), each with their own sequence specificity. A recent proteome-wide study revealed that loss of NatA in *S. cerevisiae* leads to slower cell growth, enhanced responses to stress conditions, and decreased protein stability due to defective protein–protein interactions and improper folding [31]. No specific N-terminal deacetylases have been identified thus far, and this addition is generally considered irreversible [28]. These modifications affect protein half-life through the ^Ac^N-end rule pathway that can direct proteosome-mediated degradation through proteins (recognins) that detect the acetyl group and target the acetylated molecule for polyubiquitination and subsequent destruction [29,32]. In addition, other N-terminal modifications have been reported in metazoans, such as methylation following methionine removal, which, for example, is important for controlling the affinity of RCC1 for DNA and the normal progression of mitosis [33,34]. 

Here, we have used mass spectrometry to examine the N-termini of axonemal and IFT dynein proteins from the ciliated green alga *Chlamydomonas*. We found that two distinct acetylation pathways, one of which requires the action of a methionine aminopeptidase, are used to modify most dynein components. These acetylated sites should act as degrons, and the modified dynein components are likely subject to the ^Ac^N-end rule. We also determine that several dynein proteins are processed to expose a Pro residue but not subsequently acetylated; these may provide a protein concentration set point to which other synthesized dynein components are compared, thereby leading to stoichiometric balance during holoenzyme assembly. These data raise the possibility that N-terminal acetylation affects the stability of dynein proteins and intra-dynein associations and/or potentially contributes to the maintenance of stoichiometric levels of axonemal dynein components in the cytoplasm. Thus, our data reveal further complexity in the processing pathway(s) leading to the formation of axonemal dynein holoenzymes in the cytoplasm. 

## 2. Materials and Methods

### 2.1. Computational Methods

Mining of the *Chlamydomonas reinhardtii* CC-4532 v6.1 genome for N-terminal acetylase subunits was performed using keyword and BLAST searches at Phytozome 13 (https://phytozome-next.jgi.doe.gov/ (accessed on 9 October 2023)) and the National Center for Biotechnology Information (https://blast.ncbi.nlm.nih.gov/Blast.cgi (accessed on 9 October 2023)). Predictions for the subcellular location of proteins were made using the PredAlgo (PredAlgo Main (ibpc.fr)) [35] and TargetP (TargetP 2.0—DTU Health Tech—Bioinformatic Services) [36] algorithms. Structural models of the N-terminal residues of dynein proteins were generated using the builder interface within the PyMOL molecular graphics system (version 2.0, Schrödinger, LLC). Hydrophobicity was displayed on the stick models using the PyMOL command color_h, and the electrostatic potential within the range ±5.0. *K*_b_*T*/e_c_ was calculated with the adaptive Poisson–Boltzmann solver plugin within PyMOL and used to paint the molecular surface; surface transparency was adjusted to 40%. 

### 2.2. Chlamydomonas Strains and Growth Conditions

Wild-type and the outer-arm-less mutant *oda2 Chlamydomonas reinhardtii* strains were obtained from the *Chlamydomonas* Resource Center at the University of Minnesota (https://www.chlamycollection.org/ (accessed on 24 May 2023)). For the whole cilia sample and the cilia samples used to obtain electrophoretically purified axonemal dynein HCs, *Chlamydomonas* wild-type strains CC-125 (mating type *plus*) or *oda2* (CC-2230) were grown in an acetate-containing R medium with constant aeration on a 12:12 h light:dark cycle. Cilia samples used for proteomic analysis of the entire membrane plus matrix and axoneme fractions derived from both CC-125 and its mating-type *minus* counterpart CC-124. In addition, cilia from both vegetatively growing cells and gametes induced by nitrogen starvation were used, as described previously [25].

### 2.3. Preparation of Cilia Samples for Mass Spectrometry

As described in [25], cells were harvested using low-speed centrifugation (Fiberlite F10, 1000× *g*, 7 min) and washed three times with 10 mM HEPES pH 7.5. Pellets were then resuspended in ice-cold 30 mM HEPES pH 7.5, 5 mM MgSO_4_, 1 mM dithiothreitol, and 4% (*w*/*v*) sucrose (10 mL/tube) containing protease inhibitors (Sigma–Aldrich, St. Louis, MO, USA, P9599). Subsequently, 100 μL/tube of 5.3% (*w*/*v*) CaCl_2_ was added, followed by 2 mL of 25 mM dibucaine-HCl to deciliate the cells. Deciliated cell bodies were collected using low-speed centrifugation, and the cilia-rich supernatant overlaid on a 25% sucrose cushion and spun in a swing-out rotor (2400× *g*, 10 min, Sorvall ST-8) to remove any remaining cell bodies. Isolated cilia were then demembranated with 1% (*v*/*v*) IGEPAL-CA630 detergent to yield membrane plus matrix and axoneme fractions following high-speed centrifugation (Axyspin R centrifuge, 13,500 rpm, 4 °C, 20 min). An additional whole cilia sample from vegetative CC-125 cells was prepared separately and resuspended directly in 5% SDS in 20 mM Tris.Cl pH 8.0 before being processed for mass spectrometry.

### 2.4. Peptide Identification Using High-Resolution Mass Spectrometry

Mass spectrometry of membrane plus matrix and axoneme samples from whole cilia of vegetative and gametic *Chlamydomonas* cells (strains CC-124 and CC-125) separated in a “short gel” format was performed at the University of Massachusetts Medical School Mass Spectrometry Facility and was described in detail previously [25]. 

To analyze axonemal dynein HCs, axoneme samples were electrophoresed in 4–15% SDS gradient Mini-PROTEAN TGX polyacrylamide gels (Bio-Rad, Hercules, CA, USA), stained with Coomassie blue and the HC region excised. Gel bands were destained with 40% ethanol and 10% acetic acid in water, diced, and equilibrated in 100 mM ammonium bicarbonate pH 8. Proteins from gel bands and the whole cilia sample were reduced and alkylated on Cys residues by incubating in 10 mM dithiothreitol (37 °C for 60 min) followed by 55 mM iodoacetamide (37 °C for 45 min in the dark); both made in 100 mM ammonium bicarbonate pH 8. Gel pieces were dehydrated in acetonitrile for 20 min and dried to completion. For proteolytic digestion, dehydrated gels were first resuspended in either 12.5 ng/μL trypsin (Promega porcine sequencing grade ^P^/_N_ V5113) or 12.5 ng/μL AspN (Roche Endoproteinase AspN, ^P^/_N_ 11054589001) in 100 mM ammonium bicarbonate on ice for 45 min. The excess solution was then replaced with 100 mM ammonium bicarbonate, and digestion was allowed to occur for 16 h at 37 °C with constant mixing. Peptides were extracted using two alternating washes with 100 mM ammonium bicarbonate and 50% acetonitrile in 5% formic acid, followed by two alternating washes with 100 mM ammonium bicarbonate and 100% acetonitrile. All peptide-containing solutions were then dried to completion and resuspended in 0.1% formic acid in water.

Resuspended peptides were analyzed using nanoflow ultra-high performance liquid chromatography (UPLC) coupled with tandem mass spectrometry (MS/MS) using a Dionex Ultimate 3000 RSLCnano UPLC system and Orbitrap Eclipse Tribrid mass spectrometer (Thermo Scientific). Peptides were loaded onto a 75 µm × 25 cm nanoEase *m*/*z* peptide BEH C18 analytical column (Waters Corporation), separated using a 1 h reverse-phase UPLC gradient, and directly ionized into the Orbitrap Eclipse using positive mode electrospray ionization. MS/MS data were acquired using a data-dependent Top 15 MS/MS acquisition method in the Orbitrap mass analyzer. The whole cilia sample was analyzed using higher-energy collisional dissociation. All raw data were searched against the *Chlamydomonas reinhardtii* CC-4532 proteome database v6.1 from Phytozome (https://phytozome-next.jgi.doe.gov (accessed on 27 April 2023)) using the Andromeda search engine embedded within MaxQuant v1.6.10.43 [37]. Label-free quantification was performed using MaxQuant. Variable modifications included were Met oxidation, N-terminal Met removal, methyl Lys/Arg, dimethyl Lys/Arg, and trimethyl Lys. Variable N-terminal modifications searched were acetylation, arginylation, mono-, di-, and tri-methylation, myristoylation, palmitoylation, and ubiquitination. The carbamidomethylation of Cys was included as a fixed modification. Enzyme specificity was set to “Trypsin/P” or “AspN”, the minimum peptide length was set to “5”, and the false discovery rate was set to 1% at both the protein and peptide-spectrum match levels. All other parameters were kept at default settings. The MaxQuant results were uploaded into Scaffold v5 (Proteome Software) for visualization and further analysis.

## 3. Results

### 3.1. N-Terminal Acetylases in Chlamydomonas

Seven distinct N-terminal acetylases have been identified in eukaryotes (NatA-NatH), although most modifications are performed by the NatA, NatB, or NatC complexes [27,28,38]. The NatD and NatH enzymes only acetylate histones and actin, respectively, whereas the specificity of NatE is similar to NatC. The NatF complex associates with the Golgi and usually acetylates transmembrane proteins. In general, these enzymes consist of a catalytic subunit combined with a ribosome anchoring protein and, for NatA and NatC, an auxiliary subunit whose role has yet to be fully defined. Examination of the *Chlamydomonas* genome (CC-4532 ver. 6.1.; Phytozome 13 database) using BLAST revealed putative chlorophycean orthologs for the catalytic components of six of the protein N-terminal acetylases present in metazoans and plants (Table 1). For example, *Chlamydomonas* NAT1 (Cre08.g364450) is 66% identical to human NAA10 (the catalytic subunit of the NatA complex) and this comparison gives a BLAST expect value of 4.88 × 10^−68^; similarly, human NAA20 (the catalytic component of NatB) shares 63% identity with *Chlamydomonas* NAT3 (Cre08.g383150) with an expect value of 1.84 × 10^−78^. An obvious ortholog of the actin-specific NatH enzyme present in metazoans was not identified in *Chlamydomonas* or *Arabidopsis*. In addition, we note that *Chlamydomonas* encodes numerous other proteins with acetyltransferase domains that have yet to be functionally classified.

**Figure 1:**
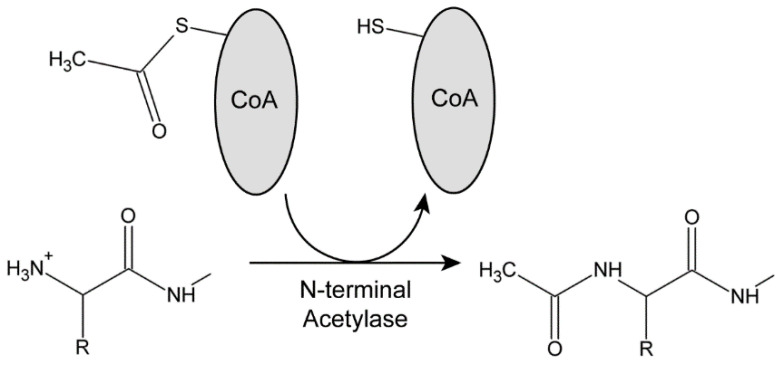
The Protein N-terminal Acetylation Reaction. Acetylases use acetyl-CoA as a source of the acetyl group that is transferred to the N-terminal amino group of the target protein. Breakage of the thioester bond in acetyl-CoA provides the energetic driving force for the reaction. N-terminal acetylation results in the removal of the +1 positive charge, and so alters the electrostatics of the protein N-terminus. To date, no specific N-terminal deacetylases have been identified.

These enzymes hydrolyze the thioester bond of acetyl-CoA in an exergonic process to provide the acetyl group and the energetic driving force for the reaction (Figure 1). Each acetylase has a distinct sequence specificity [27]. NatA requires the removal of the initiating Met residue by a methionine aminopeptidase; in *Chlamydomonas,* this is thought to be performed by MAP1A (Cre06.g279750) based on a comparison with *Arabidopsis* [39]. Subsequently, NatA acetylates exposed residues with small R groups (usually Ala, Ser, Thr, and Cys). NatB modifies the terminal Met residue directly, with specificity being determined by the residue at the M + 1 position (Asp and, more rarely, Glu, Asn, or Gln). NatC again acetylates the initial Met if it is followed by a hydrophobic or amphipathic residue (Leu, Ile, Phe, Trp, Val, Met, His, or Lys). Importantly, if a Pro residue is exposed following methionine aminopeptidase activity, it is usually not subject to N-terminal acetylation [29].

### 3.2. Acetylation of Dynein Heavy-Chain N-Termini

*Chlamydomonas* encodes fifteen axonemal dynein HCs (DHCs1-15) and the dynein motor DHC1b (DHC16) required for retrograde intraflagellar transport. To assess the modification of the N-termini of these dynein HCs, we isolated demembranated ciliary axonemes from wild-type *Chlamydomonas* cells (strain CC-125), subjected them to gel electrophoresis and excised the HC-containing band following staining with Coomassie blue. After destaining, the samples were subject to protease digestion using either trypsin, which cleaves peptide bonds to the C-terminal side of Arg and Lys residues, or the zinc metalloendopeptidase Asp-N, which cleaves peptide bonds N-terminal of Asp (and sometimes also Glu) acidic residues, followed by liquid chromatography and tandem mass spectrometry. Using this approach, we were able to determine the N-terminal status for twelve of the sixteen HCs. The other four HCs (DHC1, DHC4, DHC5, and DHC12) have Arg and/or Asp residues very close to the N-terminus, which are likely cleaved to yield peptides too small for unambiguous identification and assignment (Table 2); DHC4 and DHC12 are also “minor” dyneins present in greatly reduced amount relative to most others [40]. However, based on their N-terminal sequences and the modifications observed in other HCs, the status of these four HCs can be predicted. Our extensive mass spectrometry searches also revealed no evidence that axonemal dynein HC N-termini are subject to arginylation, myristoylation, palmitoylation, or ubiquitination.

**Figure 2:**
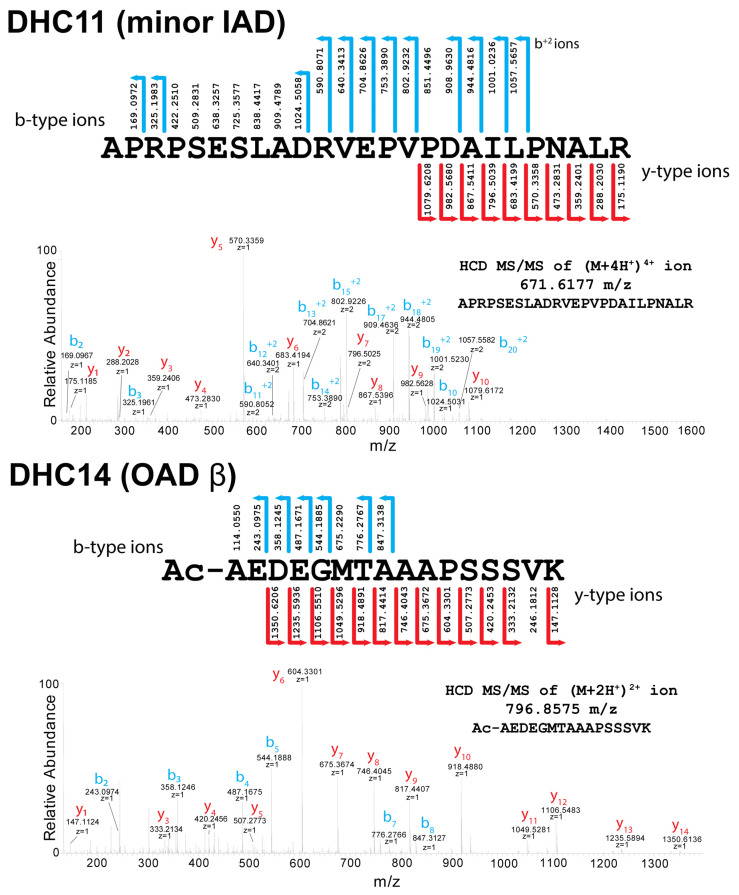
Mass Spectral Analysis of Type I and II Dynein Heavy-Chain N-termini. Annotated higher energy C-trap dissociation (HCD) MS/MS spectra and the corresponding fragment coverage with identified b- and y-type ions of N-terminal peptides from the minor inner-arm dynein DHC11 (Type II) and the outer-arm dynein β HC (DHC14; Type I). These spectra and fragment *m*/*z* values unambiguously demonstrate that the N-termini of both HCs are processed by methionine aminopeptidase to expose an Ala residue. The mass spectral data further reveal that although DHC14 is then acetylated, likely by the NatA complex, the exposed Ala residue of DHC11 is left unmodified.

*Chlamydomonas* dynein HCs fall into four distinct classes based on the nature of the N-terminal modification and the necessary processing pathway required to generate it (Table 2). Class I consists of the IFT dynein HC (DHC16), the three outer-arm HCs (DHCs13-15), and two monomeric inner-arm HCs (DHC4 and DHC7). These are all processed by methionine aminopeptidase to expose an Ala or Ser residue that is then acetylated predictably by the NatA complex. In class II, one other inner-arm HC (DHC11) is also processed to leave an exposed Ala that remains unmodified. Examples of the mass spectral data and fragment ion-coverage-comparing class I (acetylated) and II (unmodified) N-terminal peptides (from DHC14 and DHC11, respectively) are shown in Figure 2.

Class III HCs include monomeric inner-arm HCs DHC6 and DHC9 and the inner-arm I1/f 1beta HC (DHC10) and are not targets of methionine aminopeptidase; all three HCs are directly acetylated on the initiator N-terminal Met residue that is followed by Asp or Glu. Although not identified in the mass spectral data, based on their sequences, DHC1 (inner-arm I1/f 1alpha HC) and monomeric dyneins DHC5 and DHC12 also predictably belong to this group. As these class III HCs have Asp or Glu at position 2, they are likely modified by the NatB complex. The final HC group (class IV; monomeric inner-arm HCs DHC2, DHC3, and DHC8) all have Pro at position 2. For these HCs, the Met residue is removed, but the Pro residue is left unmodified; this is consistent with the known specificity of N-terminal acetylases, which do not readily recognize exposed Pro residues [28]. 

The twelve HCs belonging to Classes I and III start with acetylated Ala, Met, or Ser residues which represent ^Ac^N-degrons, and these proteins are potentially subject to the ^Ac^N-end rule pathway, which targets select proteins for regulated destruction. Notably, the four dynein HCs that are not acetylated are all monomeric motors that cluster around the radial spokes. Two (DHC3 and DHC11) are “minor” dyneins whose axonemal location has yet to be determined [41]. The others form dyneins d (DHC2) and e (DHC8), located on the ciliary tip side of radial spoke 2 (dynein e) and the truncated spoke 3s (dynein d) [42].

**Figure 3:**
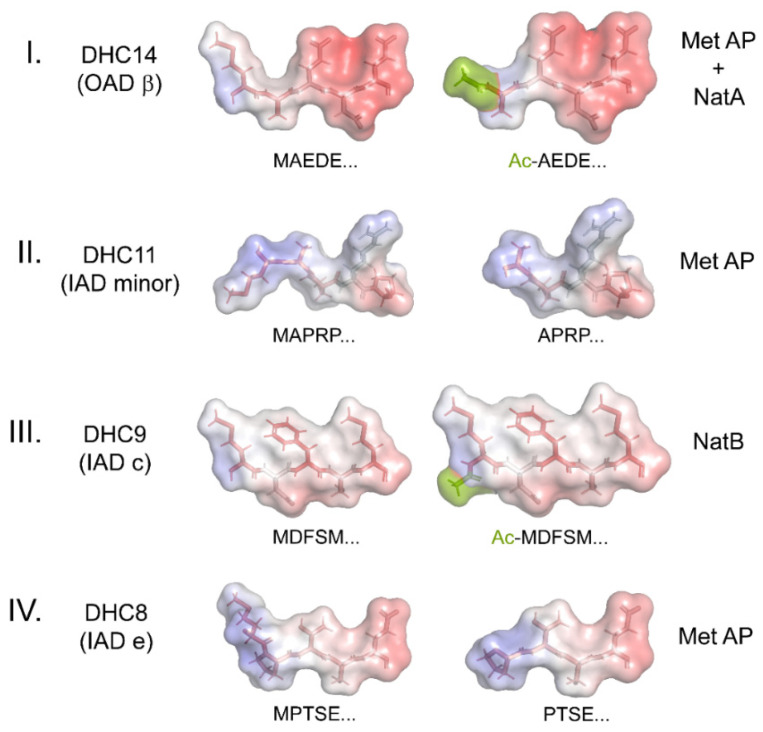
Structural Models of Dynein Heavy-Chain N-termini. The models illustrate the changes in structure, hydrophobicity, and electrostatics that occur upon the N-terminal processing of dynein HCs using methionine aminopeptidase (Met AP) and/or the NatA/NatB acetylase complexes. The N-terminal five residues for each HC were built as a β-strand using the PyMOL builder interface, and the stick structures were colored by hydrophobicity (hydrophilic, white; hydrophobic, red). The semi-transparent molecular surface is painted to reveal the electrostatic potential from −5.0 (red) to +5.0 (blue). *K*_b_*T*/e_c_ was calculated using the adaptive Poisson–Boltzmann solver within PyMOL. The acetyl groups added to the termini of HC classes I and III are a split-pea color.

Methionine removal and/or acetylation have substantial impacts on the charge, structure, electrostatics, and hydrophobicity of these N-terminal regions of the various HCs. Structural models of the N-termini of four HCs before and after processing, one from each HC class, are shown in Figure 3. These models clearly reveal that N-terminal modifications have significant effects that will likely lead to altered assembly and/or interaction characteristics. They also emphasize that the various dyneins have N-termini with very distinct topologies, hydrophobicity, and electrostatic properties.

This analysis of the N-termini of dynein HC components led us to examine available cryogenic electron microscopy-based reconstructions of dynein particles and doublet microtubule-associated structures (e.g., [43,44]) to try and further understand the structural context and consequences of HC N-terminal processing. In some cases, these segments are absent from the structures as presented. Whether this reflects heightened flexibility and thus low electron density in the maps for certain regions and/or other factors, such as a resolution too low to recognize individual acetyl groups, is unclear. 

**Figure 4:**
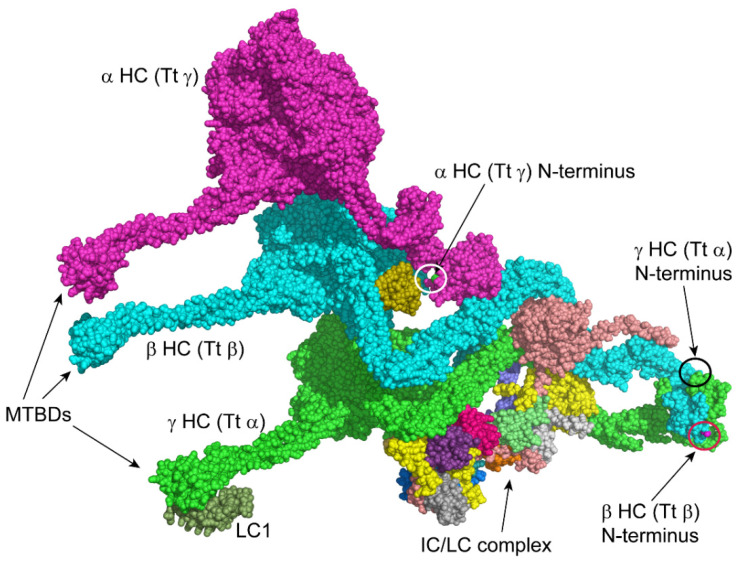
Accessibility of Heavy-Chain N-termini in the Outer Dynein Arm. The surface of the multi-component ~2 MDa outer-arm dynein from *Tetrahymena thermophila* (PDB 7K5B, [44]) is shown with each individual chain in a different color. For historical reasons, some *Tetrahymena* outer-arm HCs have different names to orthologs in *Chlamydomonas* [45]. To avoid confusion with the rest of the text, the individual HCs have been labeled using the *Chlamydomonas* name with the *Tetrahymena* name in parentheses, e.g., α HC (Tt γ). The HC microtubule-binding domains (MTBDs) are at *left*. The α HC (Tt γ) N-terminus (cyan; white circle) is buried, whereas those of the β (Tt β) (purple; red circle) and γ (Tt α) (green; black circle) HCs are accessible in the holoenzyme: although the terminal four residues are missing from the γ (Tt α) HC density map, the approximate location of this N-terminus is indicated by the black circle. Once incorporated into the axoneme, these β and γ HC termini will be near the doublet microtubule A-tubule. However, another cryo-EM reconstruction (PDB 7MOK, [46]) suggests that these termini would point away from the outer-arm docking complex and microtubule surface, so they are unlikely to form part of the binding site with the docking complex. As the motor units of one outer dynein arm overlap the basal region of the neighboring arm, potentially, these termini are buried at the interface between adjacent outer dynein arms. This figure was generated using PyMOL.

In a structure of the *Tetrahymena thermophila* outer arm (PDB 7K5B; [44]), the N-terminal region of the β HC is of relatively low local resolution, and the side chains cannot be fitted with confidence in the electron density (Figure 4); our data from *Chlamydomonas* indicate that in this organism the Met is removed and the exposed Ala acetylated. Although still accessible in the isolated outer arm, when incorporated into the axonemal superstructure, this β HC N-terminus is likely buried at the interface between adjacent dynein arms. The N-terminus is also present in the *Tetrahymena* γ HC structure, which, for complex historical nomenclature reasons [45], is the equivalent of the *Chlamydomonas* α HC; here, the terminus is again processed by methionine aminopeptidase and acetylated. This N-terminus would be readily accessible in monomeric α HCs prior to their assembly into multimeric complexes. However, it becomes sequestered and relatively inaccessible once the HC has been incorporated into the outer-arm holoenzyme that forms in the cytoplasm, as it is essentially buried between two HCs and one of the thioredoxin-like LCs (Figure 4). Thus, in the assembled state, any N-terminal degron on this HC would be hidden. Although the terminal four residues are missing from the γ (Tt α) HC density map, this terminus is likely exposed in the isolated dynein particle, as is the β HC N-terminus.

### 3.3. Acetylation of Other Axonemal and IFT Dynein Components

To gain insight into N-terminal modifications that might occur within other dynein components, we reanalyzed our previously published *Chlamydomonas* ciliary proteome datasets focusing on the twenty-four trypsin-digested ciliary axoneme and membrane-plus-matrix fractions from vegetative and gametic wild-type cells of both mating types [25] (see *Chlamydomonas* Ciliary Proteins (chlamyfp.org)) and an additional wild-type whole cilia sample digested with both trypsin and AspN. Although we could identify the N-terminal status of numerous components, there were several for which we were unable to determine the N-terminal residue (Table 3). This likely reflects tryptic cleavage sites close to the N-termini that result in peptides too short to provide an entirely unique amino acid sequence for unambiguous identification or possibly low abundance (copy number) of individual dynein proteins relative to the high abundance of some other ciliary components. The latter factor can lead to lower sequence coverage.

**Figure 5:**
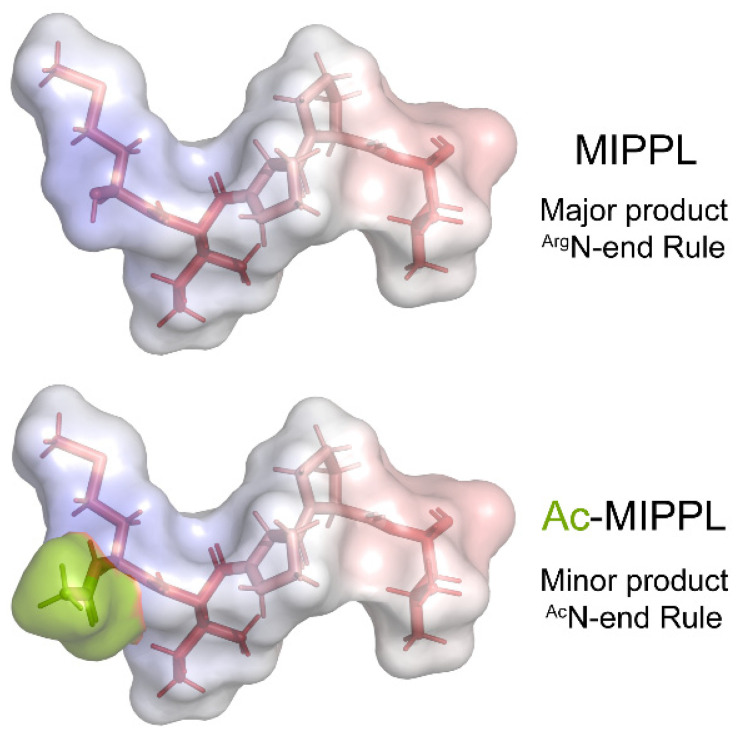
Structural Models of the Two N-Termini Identified for IDA4/p28. The majority of IDA4/p28 was found to have an unmodified terminus that conforms to the Type 2 consensus of the ^Arg^N-end rule pathway. However, in one sample, an acetylated form was also clearly present, although at an intensity much less than the unmodified version. This acetylated form represents an ^Ac^M-Φ-degron subject to the ^Ac^N-end rule pathway. Thus, two different forms of IDA4/p28 are present in axonemes. Whether they occur within distinct monomeric inner-arm dyneins remains uncertain. The N-terminal five residues of both forms of IDA4/p28 were built as a β-strand using the PyMOL builder interface, and the stick structures were colored by hydrophobicity (hydrophilic, white; hydrophobic, red). The semi-transparent molecular surface is painted to reveal the electrostatic potential from −5.0 (red) to +5.0 (blue). *K*_b_*T*/e_c_ was calculated using the adaptive Poisson–Boltzmann solver within PyMOL. The acetyl group is colored a split-pea color.

Most non-HC dynein components whose N-termini were identified begin with either acetylated Ala, Met, or Ser residues, as do the predicted termini for the remaining six components. Unmodified Pro residues were found for the N-termini of outer-arm dynein IC1 and for DC2 of the outer-arm docking complex. The novel actin-related protein NAP that can functionally substitute for conventional actin in several monomeric inner-arm dyneins [47] has an unmodified Thr at the N-terminus. One other unusual terminus was identified in IDA4/p28, which is a dimeric component of monomeric inner arms a, c, and d (containing DHCs 6, 9, and 2, respectively) and the minor dyneins DHC11 and DHC12 [40]; this protein is required for the assembly of these motors within the axonemal superstructure [48,49]. IDA4/p28 has an unmodified Met residue at the N-terminus followed by Ile-Pro-Pro-Leu; if acetylated, this would represent an ^Ac^M-Φ degron subject to the ^Ac^N-end rule pathway. However, we obtained only a single acetylated peptide in just one of twenty-four cilia samples at an intensity at least 10-fold less than the unmodified form in the same sample (Figure 5); this observation was repeated in an independently prepared whole cilia sample. Thus, nearly all axonemal IDA4/p28 is not acetylated. One possibility that remains to be tested is that the acetylated form may be associated with only a subset of the five inner-arm dyneins that contain this component.

Intriguingly, the unmodified IDA4/p28 N-terminus does, however, fit the consensus for primary destabilizing residues subject to the ^Arg^N-end rule pathway, in which either Met removal is followed by the arginylation of exposed Asp/Glu residues (Type 1) or the N-terminal Met is followed by a residue with a bulky hydrophobic side chain such as Ile, Leu, Phe, Trp, or Tyr (Type 2) [29]. The recognin that detects these ^Arg^N-end rule termini in *S. cerevisiae* and mammals is Ubr1 [50]. The *Chlamydomonas* genome encodes an E3 ubiquitin ligase with a UBR-like zinc finger domain (Cre02.g800188) that is predicted by both the TargetP and PredAlgo algorithms to be cytosolic (note that the location annotation in the latest version (ver. 6.1) of the *Chlamydomonas* genome in Phytozome v.13 is incorrect). In addition, *Chlamydomonas* expresses another E3 ligase termed PRT1 that has been found to recognize unmodified destabilizing residues in plants [51]. Thus, one or both of these E3 ligases might potentially target the terminus of unmodified IDA4/p28.

**Figure 6:**
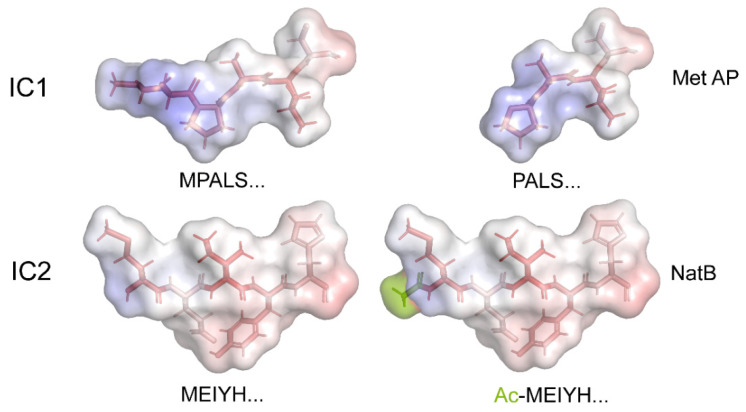
Structural Models of the N-Termini of Outer-Arm Dynein IC1 and IC2. The termini of IC1 and IC2 before and after processing by methionine aminopeptidase (Met AP) and the NatB acetylase (NatB), respectively, are shown. The structure, hydrophobicity, and electrostatics are all changed via these modifications. As these regions are missing from available cryo-EM structures, the N-terminal residues were built as a β-strand using the PyMOL builder interface, and the stick structures were colored via hydrophobicity (hydrophilic, white; hydrophobic, red). The molecular surface is painted to reveal the electrostatic potential from −5.0 (red) to +5.0 (blue) *K*_b_*T*/e_c_ calculated using the adaptive Poisson–Boltzmann solver within PyMOL. The acetyl group added to IC2 is painted a split-pea color.

In *Chlamydomonas*, the fully formed outer dynein arm is constructed from a series of independently assembling modules that, in addition to three HCs, include an intermediate chain/light chain complex (consisting of IC1, IC2, LC2, LC6, LC7a, LC7b, LC8, LC9, and LC10) and a docking complex (of DC1, DC2, and the calmodulin paralog DC3); the latter trimeric structure can incorporate into the axoneme in the absence of the other components [52]. Both these multimeric sub-complexes can exist independently within the cytoplasm. Although most of these proteins are N-terminal acetylated, it is notable that a single component from both the IC/LC and docking complexes (IC1 and DC2, respectively) are processed by methionine aminopeptidase to yield an unmodified Pro residue at the terminus that would not be subject to the ^Ac^N-end rule; a single low-intensity, low-confidence DC2 peptide terminating in Met was also observed. Thus, their synthesis might provide a set point against which levels of all other components of these sub-complexes could be measured or compared to ensure the appropriate cytosolic stoichiometries; a comparison of the original and modified N-termini IC1 and IC2 is shown in Figure 6.

## 4. Discussion

### 4.1. Integrating N-Terminal Acetylation within the Axonemal Dynein Assembly Pathway

The scale of axonemal dynein motor assembly in ciliated cells necessitates a large array of cytoplasmic factors (DNAAFs; currently, nineteen are recognized [17]) that are specifically needed in addition to the usual chaperones, prefoldins, ribosomal components, and other complexes generally involved in building and folding cellular proteins [17,18]. It takes about 14–15 min of ribosome activity to assemble a single ~540 kDa dynein HC encoded by a ~5 μm long mRNA at a rate of ~5 residues per second [19]. In mammalian cells, dynein formation occurs in cytosolic membrane-less condensates, which contain both assembly factors and dynein components; these can be considered dynein assembly factories [53,54]. The biosynthetic burden of axonemal dynein formation is considerable as a single ciliated mammalian tracheal epithelial cell might have three hundred cilia and need to assemble millions of dyneins with a total mass in the TDa range. Even in the biciliate alga *Chlamydomonas*, over fifty-thousand axonemal dyneins HCs are needed [19]. 

Axonemal dynein assembly factors may be classified into several groups, although the precise role of many remains uncertain [17]. For example, some are PIH proteins that recruit heat-shock proteins and interact with specific scaffolding factors to generate variant forms of the R2TP complex that contain the RuvBL1 and RuvBL2 AAA ATPases [55,56]. Different dynein types require distinct PIH proteins [57,58], and there is also evidence that distinct assembly proteins, including variant R2TP scaffolds [59] and chaperone recruiters, act sequentially during HC assembly, e.g., [60]. Other assembly factors such as WDR92 (DNAAF10), HEATR2 (DNAAF5), and LRRC6 (DNAAF11) provide protein–protein interaction domains, e.g., [58,61,62]. Furthermore, many of the assembly factors contain large regions that are inherently disordered [63] and likely only attain a stable fold when bound to regions of nascent-growing HCs. As a consequence, the roles of many DNAAFs and numerous features of the complex holoenzyme assembly pathway(s) remain to be clarified.

The multiple pathways of N-terminal processing we have revealed here for various dynein components uncover yet more complexity in this process. As methionine removal and N-terminal acetylation often occur during translation, this predicts that methionine aminopeptidase and both NatA and NatB complexes likely reside within the cytoplasmic assembly factories for axonemal dyneins. Predictably, once generated, the HC N-terminal regions and other components interact to form stable associations, even though the remainder of the molecules are still being synthesized. This inter-HC association or interaction with other dynein components may protect them from targeted degradation. As each HC mRNA may have a large number of ribosomes simultaneously translating new proteins, N-terminal processing is likely to occur under conditions where they experience a high concentration of the cognate interaction site, thereby driving the assembly process.

### 4.2. ^Ac^N-Degrons as a Potential Mechanism to Direct Dynein Subunit Stoichiometry 

One key feature of axonemal dynein assembly is the synchronized expression of the various components. Studies in *Chlamydomonas* revealed that dynein gene transcription is rapidly and coordinately upregulated in response to deciliation [64,65]. However, how cells balance translation from dynein mRNAs, which can have very different lengths with very different numbers of attached ribosomes, and then adjust for the difference in time taken to build a HC (about 14 min or so) vs. a 10 kDa LC (perhaps 20 s) is much less certain. Fractionation of cytoplasmic extracts reveals that most dynein components are present in assembled sub-complexes [61,66]. For example, outer-arm dynein ICs are found in a multi-subunit IC/LC complex that is either associated with its HC partners or free in the cytoplasm [66]. What is not observed for the axonemal dynein-specific subunits of this IC/LC complex are individual proteins migrating as monomers during fractionation of cytoplasm. Our data indicate that one component of this complex, IC1, has an N-terminal Pro, whereas all others are acetylated. As it is generally considered that N-acetylated proteins are not targeted for degradation once assembled into multi-protein complexes, this provides a potential mechanism to balance the amounts of available IC/LC complex proteins. Any component of the IC/LC complex made in amounts greater than IC1 would be subject to the ^Ac^N-end rule and degraded. A similar prediction can be made about the trimeric outer-arm docking complex based on the non-acetylated Pro residue at the N-terminus of DC2 and the acetylated Ala on both DC1 and DC3. As dynein components are synthesized and assembled in discrete condensates, the formation of the IC/LC complex would allow it to rapidly bind the N-terminal regions of the outer-arm dynein β (DHC14) and γ (DHC15) HCs as they are being synthesized, thereby further protecting them from co-translational recognin binding. Indeed, the apparent super-stoichiometric synthesis of the IC/LC complex (e.g., [66]) may provide a mechanism to ensure that nascent HCs, which are energetically expensive to synthesize, are not destroyed unnecessarily. It is intriguing that four monomeric inner-arm dynein HCs are not acetylated at the N-terminus, which suggests that these might represent setpoints to which other inner-arms or inner-arm components are compared. This may be particularly important during ciliary assembly for adjusting the complex stoichiometries of these dyneins, some of which are differentially located along the axonemal length. 

In contrast, a series of outer-arm dynein LCs that associate directly with HCs and potentially act as regulatory factors (LCs1, 3, 4, and 5) all have acetylated Ala at the N-terminus and are found as either HC-bound or as monomers [66]. Given that these proteins are small, in the 14–22 kDa range, their synthesis is fast, and they may be subject to rapid turnover if a HC target is unavailable.

### 4.3. Recognition of Dynein ^Ac^N-Degrons

Given the large number of assembly factors already identified as specifically functioning in axonemal dynein formation [17], the sequestration of dynein assembly within dedicated phase-separated compartments or factories [53], combined with the generation of axonemal dynein ^Ac^N-degrons demonstrated here, there might be one or more E3 ubiquitin ligase(s) involved as dynein-specific ^Ac^N-recognins that help maintain appropriate subunit stoichiometry and/or function in axonemal dynein protein quality control. Indeed, it seems likely that mechanisms for protein quality control and stoichiometry maintenance would be located at sites of active dynein protein synthesis and particle formation. Although no proteins have been experimentally linked to this type of activity to date, it is intriguing that *Chlamydomonas* encodes a motility-associated E3 ubiquitin ligase termed MOT4 (Cre06.g278187) [67]; orthologs of this protein are present only in organisms with motile cilia and, for example, are specifically absent in the nematode *Caenorhabditis elegans* that only assembles immotile sensory cilia, and from all angiosperms, which completely lack cilia [67]. Many other proteins identified by comparative genomics with a similar phylogenetic signature [67] were either already known or subsequently found to play a key role(s) in the assembly and/or function of specifically motile cilia, e.g., MOT48, which is an axonemal dynein assembly factor involved in the recruitment of heat-shock proteins [57]. 

MOT4 consists of an N-terminal domain structurally related to Shu2 from *S. cerevisiae* (RMSD = 2.57 Å over 59 residues), which binds Zn^2+^ through a Cys-X-Cys-(X)_n_-Cys-X-His motif and forms part of a tetrameric complex involved in Rad51 filament formation during homologous recombination and dsDNA break repair (PDB 5XYN; [68]); sequence analysis and modeling revealed that MOT4 contains a perfect copy of this Zn^2+^ binding motif. This domain is followed by two Zn^2+^-binding RING domains that likely ubiquitinate substrates and are interconnected by an alpha helix; MOT4 terminates in an extended unstructured region, as do many dynein assembly factors. This protein is not present in our cilia proteome datasets [25] and is also absent from other available *Chlamydomonas* ciliary proteomes (see the extensive compilation at *Chlamydomonas* Ciliary Proteins (chlamyfp.org)). Thus, although cilia do contain several E3 ligases and ubiquitination does occur in the ciliary compartment [69], it is unlikely that MOT4 is involved due to its subcellular localization, i.e., its targets are almost certainly cytosolic. In the future, it will be interesting to test whether MOT4 localizes to cytoplasmic sites of dynein assembly and might act as an axonemal dynein-specific E3 ligase.

### 4.4. A Single Inner-Arm Dynein Protein Is Subject to the ^Arg^N-End Rule Pathway

The N-end rule pathway includes several distinct branches. As detailed above, most dynein proteins are N-terminal acetylated and potentially processed by the ^Ac^N-end rule via dedicated recognins targeting them for proteosomal destruction. However, a second branch, the ^Arg^N-end pathway, involves distinct recognins binding to either Type 1 or Type 2 N-termini [29]. Type 1 termini are generated through processing via methionine aminopeptidase to yield an exposed Asp or Glu residue that is then arginylated by a dedicated arginyltransferase; in *Chlamydomonas,* this activity is performed by ATE1 encoded at Cre13.g580600. In some cases, a deaminase acts on exposed Asn or Gln residues to convert them to the corresponding acid prior to arginylation. In contrast, Type 2 termini retain the initial Met when it is followed by a bulky hydrophobic residue. Both these motifs can act as degrons. 

Among dynein proteins, one inner-arm component stands out as unusual. Rather than being fully acetylated, IDA4/p28 is the only component that was found to normally terminate in an unmodified Met, followed by a bulky hydrophobic residue forming an M-Φ degron that is potentially processed via the ^Arg^N-end rule pathway. Only a very minor amount of IDA4/p28 was found acetylated on the N-terminal Met. Thus, in axonemes, there are two distinct forms of this protein that are subject to different branches of the N-end rule. This N-terminal sequence (Met-Ile-Pro-Pro-) is highly conserved in IDA4/p28 orthologs from chlorophyte algae to vertebrates, implying that it plays a significant functional role. It is puzzling that this dimeric protein, which is part of only a subset of monomeric inner-arms, where it binds both the HC and actin [40,70], should exhibit this distinctive binary N-terminal property. Potentially, the acetylated version may be associated with a subset of the five inner-arm dyneins that contain IDA4/p28—perhaps the DHC11 and/or DHC12 minor dyneins that are only present in the very low amounts [40].

It has been noted previously that the nematode *Caenorhabditis elegans*, which completely lacks axonemal dyneins, expresses a protein DYLA-1 (UniProt accession Q95YA5) ~40% identical to IDA4/p28, leading to the suggestion that IDA4/p28 orthologs may have additional role(s) unrelated to their function in the axoneme [71]. However, this nematode protein does not have the IDA4/p28 M-Φ degron, so it seems unlikely that this other potential function specifically requires the ^Arg^N-end rule pathway; indeed, DYLA-1 is most likely processed to yield an N-terminal acetylated Ala residue. Furthermore, in the green lineage, IDA4/p28 is present in the ciliated algae and in mosses, which have ciliated zoospores, but homologous sequences are absent from all aciliate plants, again suggesting a role confined to cilia. This is also consistent with the phenotype of IDA4/p28 intron splicing mutants in *Chlamydomonas,* which are reported to only exhibit a defective motility phenotype [49].

## 5. Conclusions

In conclusion, we describe here the variable N-terminal modifications that occur on axonemal dynein proteins within the cytoplasm of *Chlamydomonas*. In addition, we predict the multiple processing pathways required and detail alterations in N-terminal charge, topology, and hydrophobicity. These modifications are potentially involved in the control of dynein protein interactions, quality control, and the regulation of subunit availability in the cytoplasm. Our data identify and illustrate yet more complexity in the intricate assembly pathway(s) that lead to axonemal dynein formation in cytoplasm.

## Figures and Tables

**Table 1:** Putative N-terminal Acetylase Catalytic Subunits in *Chlamydomonas*.

N-Terminal Acetylase Complex	Acetylase Catalytic Subunit			*Chlamydomonas* Gene Identifier	% Identity (Expect Value)		
	Human	*Arabidopsis*	*Chlamydomonas*		*Chlamydomonas*/Human	*Arabidopsis* /Human	*Chlamydomonas/Arabidopsis*
NatA	NAA10	NAA10	NAT1	Cre08.g364450	66% (4.88 × 10^−68^)	67% (1.33 × 10^−70^)	76% (1.02 × 10^−84^)
NatB	NAA20	NAA20 (NAT3)	NAT3	Cre08.g383150	63% (1.84 × 10^−78^)	59% (3.38 × 10^−74^)	64% (4.64 × 10^−86^)
NatC	NAA30	ATMAK3	NAT6	Cre14.g623800	55% (1.39 × 10^−53^)	57% (4.00 × 10^−55^)	61% (3.51 × 10^−59^)
NatD	NAA40	NAA40	NAT24	Cre10.g431450	33% (6.62 × 10^−26^)	34% (1.25 × 10^−30^)	30% (4.59 × 10^−14^)
NatE	NAA50	NAA50	NAT11	Cre02.g101850	46% (9.06 × 10^−41^)	55% (2.20 × 10^−54^)	48% (1.32 × 10^−51^)
NatF	NAA60	NAA60	NAT29	Cre13.g572150	29% (1.04 × 10^−12^)	28% (1.18 × 10^−17^)	40% (8.44 × 10^−31^)
NatH	NAA80	----	----	----	----	----	----

**Table 2:** N-terminal modification patterns of dynein heavy chains ^#^.

Class	HC Protein (Dynein)	N-Terminal Sequence	Identified N-Terminal Residue	Predicted Terminus	Processing Requirements
I	DHC4 (IAD minor)	MSTSR…	----	Ac-Ser	Met AP + NatA
I	DHC7 (IAD g)	MASRE…	Ac-Ala	-----	Met AP + NatA
I	DHC13 (OAD α)	MSIFW…	Ac-Ser	-----	Met AP + NatA
I	DHC14 (OAD β)	MAEDE…	Ac-Ala	-----	Met AP + NatA
I	DHC15 (OAD γ)	MALDN…	Ac-Ala	-----	Met AP + NatA
I	DHC16 (IFT)	MSSDS…	Ac-Ser	-----	Met AP + NatA
II	DHC11 (IAD minor)	MAPRP…	Ala	-----	Met AP
III	DHC1 (IAD I1/f 1α)	MDRRL…	----	Ac-Met	NatB
III	DHC5 (IAD b)	MDRNR…	----	Ac-Met	NatB
III	DHC6 (IAD a)	MDWDD…	Ac-Met	-----	NatB
III	DHC9 (IAD c)	MDFSM…	Ac-Met	-----	NatB
III	DHC10 (IAD I1/f 1β)	MEPGD…	Ac-Met	-----	NatB
III	DHC12 (IAD minor)	MEPQD…	----	Ac-Met	NatB
IV	DHC2 (IAD d)	MPGVA…	Pro	-----	Met AP
IV	DHC3 (IAD minor)	MPTEL…	Pro	-----	Met AP
IV	DHC8 (IAD e)	MPTSE…	Pro	-----	Met AP

^#^ Ac, acetyl; IAD, inner-arm dynein; IFT, intraflagellar transport; Met AP, methionine aminopeptidase; Nat, N-terminal acetylase; OAD, outer-arm dynein.

**Table 3:** N-terminal acetylation patterns of other dynein components ^#^.

Dynein	Gene Symbol ^†^	Protein	N-Terminal Sequence	Identified N-Terminal Residue	Predicted N-Terminal Residue	Processing Requirements
OAD	*DIC1*	IC1	MPALS…	Pro	----	Met AP
	*DIC2*	IC2	MEIYH…	Ac-Met	----	NatB
	*DLU1*	LC1	MAKAT…	Ac-Ala	----	Met AP + NatA
	*DLT2*	LC2	MDDMP…	Ac-Met	----	NatB
	*DLX1*	LC3	MAAGL…	Ac-Ala	----	Met AP + NatA
	*DLE1*	LC4	MAAKV…	----	Ac-Ala	Met AP + NatA
	*DLX2*	LC5	MAFIT…	Ac-Ala	----	Met AP + NatA
	*DLL2*	LC6	MADEK…	Ac-Ala	----	Met AP + NatA
	*DLR1*	LC7a	MVDIA…	Ac-Val	----	Met AP + NatA
	*DLR2*	LC7b	MSDIE…	Ac-Ser	----	Met AP + NatA
	*DLL1*	LC8	MASGS…	Ac-Ala	----	Met AP + NatA
	*DLT1*	LC9	MEDDT…	Ac-Met	----	NatB
	*DLL3*	LC10	MAEQQ…	Ac-Ala	----	Met AP + NatA
	*DCC1*	DC1	MAQKS…	Ac-Ala	----	Met AP + NatA
	*DCC2*	DC2	MPSAD… ^‡^	Pro	----	Met AP
	*DLE3*	DC3	MASAA…	Ac-Ala	----	Met AP + NatA
	*DOI1*	LIS1	MSAEV…	Ac-Ser	----	Met AP + NatA
IAD I1/f ^$^	*DIC3*	IC140	MEDAS…	----	Ac-Met	NatB
	*DIC4*	IC138	MSDQK…	----	Ac-Ser	Met AP + NatA
	*DII6*	IC97	MAPKD…	----	Ac-Ala	Met AP + NatA
	*DII7*	FAP120	MDGEE…	Ac-Met	----	NatB
	*DLT3*	TCTEX1	MEGVD…	Ac-Met	----	NatB
	*DLT4*	TCTEX2b	MAEAA…	Ac-Ala	----	Met AP + NatA
	*DAU1*	ODA7	MCACM…		Ac-Cys	Met AP + NatA
IAD	*DII4*	Actin	MADEG…	Ac-Ala	----	Met AP + NatA
(monomeric)	*DII5*	NAP	MTSGL…	Thr	----	Met AP
	*IDA4*	p28	MIPPL…	Met Ac-Met ^@^	--- ---	---- NatB
	*DLE2*	Centrin	MSYKA…	Ac-Ser	----	Met AP + NatA
	*DII2*	p38	MATLT…	Ac-Ala	----	Met AP + NatA
	*DII3*	p44	MATLV…	Ac-Ala	----	Met AP + NatA
IFT ^$^	*DIC6*	D1bIC1	MSDYE…	----	Ac-Ser	Met AP + NatA
	*DIC5*	D1bIC2	MQEVP…	Ac-Met	----	NatB
	*DLI1*	D1bLIC	MAAPA…	Ac-Ala	----	Met AP + NatA

^#^ IAD, inner-arm dynein; IFT, intraflagellar transport; NAP, novel actin-related protein; OAD, outer-arm dynein. ^$^ Entries for components shared with outer-arm dynein are not repeated. ^†^ Gene names based on [45] are shown. ^‡^ This N-terminal sequence (from genome v5.6) has been experimentally confirmed. The N-terminal sequence shown in genome CC-4532 v6.1 is incorrect due to a missing intron and frame shift. ^@^ An acetylated Met residue for IDA4/p28 was identified in only one out of twenty-four samples at an intensity less than 10% that of the unmodified form in the same sample. Only the unmodified form was found in the other samples. Analysis of an independent wild-type cilia sample also found only a small fraction of the acetylated form in both AspN and tryptic digests.

## Data Availability

Mass spectral data for whole cilia and electrophoretically isolated dynein heavy chains are available at (https://datadryad.org/stash/dataset/doi:10.5061/dryad.mw6m90635 (accessed on 3 October 2023)). Our previous mass spectral data for *Chlamydomonas* ciliary proteins [25] are available at (https://doi.org/10.5061/dryad.fn2z34txn (accessed on 10 August 2022). *Chlamydomonas* sequence data are available at Phytozome 13 (https://phytozome-next.jgi.doe.gov/ (accessed on 18 October 2023)).
